# Short-term outcomes of robot-assisted versus video-assisted thoracoscopic surgery for non-small cell lung cancer patients with neoadjuvant immunochemotherapy: a single-center retrospective study

**DOI:** 10.3389/fimmu.2023.1228451

**Published:** 2023-07-11

**Authors:** Hanbo Pan, Ningyuan Zou, Yu Tian, Hongda Zhu, Jiaqi Zhang, Weiqiu Jin, Zenan Gu, Junwei Ning, Ziming Li, Weicheng Kong, Long Jiang, Jia Huang, Qingquan Luo

**Affiliations:** ^1^ Department of Thoracic Surgical Oncology, Shanghai Lung Cancer Center, Shanghai Chest Hospital, Shanghai Jiao Tong University School of Medicine, Shanghai, China; ^2^ Department of Thoracic Surgery, Shanghai Tongren Hospital, Shanghai Jiao Tong University School of Medicine, Shanghai, China; ^3^ Department of Oncology, Shanghai Lung Cancer Center, Shanghai Chest Hospital, Shanghai Jiao Tong University School of Medicine, Shanghai, China; ^4^ Department of Thoracic Surgery, Putuo District People’s Hospital, Zhejiang, China

**Keywords:** non-small cell lung cancer, robot-assisted thoracoscopic surgery, video-assisted thoracoscopic surgery, neoadjuvant immunochemotherapy, perioperative outcomes, recurrence-free survival

## Abstract

**Background:**

Neoadjuvant immunochemotherapy has been increasingly applied to treat non-small cell lung cancer (NSCLC). However, the comparison between robotic-assisted thoracoscopic surgery (RATS) and video-assisted thoracoscopic surgery (VATS) in the feasibility and oncological efficacy following neoadjuvant immunochemotherapy is scarce. This study aims to assess the superiorities of RATS over (VATS) concerning short-term outcomes in treating NSCLC patients with neoadjuvant immunochemotherapy.

**Methods:**

NSCLC patients receiving RATS or VATS lobectomy following neoadjuvant immunochemotherapy at Shanghai Chest Hospital from 2019 to 2022 were retrospectively identified. Baseline clinical characteristics, perioperative outcomes, and survival profiles were analyzed.

**Results:**

Forty-six NSCLC patients with neoadjuvant immunochemotherapy were included and divided into the RATS (n=15) and VATS (n=31) groups. The baseline clinical characteristics and induction-related adverse events were comparable between the two groups (all p>0.050). The 30-day mortality in the RATS and VATS groups were 0% and 3.23%, respectively (p=1.000). Patients undergoing RATS were associated with reduced surgical-related intensive unit care (ICU) stay than those receiving VATS (0.0 [0.0-0.0] vs. 0.0 [0.0-1.0] days, p=0.026). Moreover, RATS assessed more N1 LNs (6.27 ± 1.94 vs 4.90 ± 1.92, p=0.042) and LN stations (3.07 ± 1.03 vs 2.52 ± 0.57, p=0.038) compared with VATS. By comparison, no difference was found in surgical outcomes, pathological results, and postoperative complications between the RATS and VATS groups (all p>0.050). Finally, RATS and VATS achieved comparable one-year recurrence-free survival (82.96% vs. 85.23%, p=0.821) and the timing of central nervous system, LN, and bone recurrences (all p>0.050).

**Conclusion:**

RATS is safe and feasible for NSCLC patients with neoadjuvant immunochemotherapy, reducing surgical-related ICU stay, assessing increased N1 LNs and stations, and achieving similar survival profiles to VATS.

## 1 Introduction

Nowadays, non-small cell lung cancer (NSCLC) remains one of the most frequently diagnosed malignancies and the primary contributors to cancer-related death worldwide (1). However, despite undergoing a curative-intent surgical resection, most patients with locally advanced disease (stage IIIA-IIIB) subsequently experience tumor recurrence, mainly at distant locations, resulting in a poor prognosis with 5-year overall survival (OS) rate of less than 30% (2, 3). Unfortunately, the additional application of neoadjuvant or adjuvant chemotherapy merely leads to an improvement of about 5% in the 5-year OS rate (4). Over recent years, immunotherapy has revolutionized the treatment of NSCLC, and the oncological efficacy of immune checkpoint inhibitors (ICIs) has been well-established in patients with stage IV disease. Given this, numerous studies have further investigated the value of neoadjuvant ICIs treatment alone or with chemotherapy for resectable (stage IB-IIIA) and potentially resectable (stage IIIB) NSCLC, indicating the favorable safety and oncological efficacy of this promising therapy strategy (5–11).

Surgical resection remains the ultimate curative treatment for NSCLC if complete resection is feasible, with lobectomy remaining the gold standard. Video-assisted thoracoscopic surgery (VATS) has been widely applied, and its safety, feasibility, and oncological efficacy are well-established for early-stage and advanced NSCLC with or without neoadjuvant chemotherapy. Compared with traditional thoracotomy, VATS is associated with less surgical trauma, reduced surgical-related pain, and fewer postoperative complications (12–15). However, patients receiving neoadjuvant ICIs treatments are frequently associated with dense adhesions and fibrosis in the chest cavity, especially those with notable therapy responses, making lung resection more technically demanding (16, 17). Given this, traditional thoracotomy is still the most common surgical approach for this group of patients, with growing interest in the application of minimally invasive surgery (MIS). Several recent publications have assessed the safety and feasibility of VATS following neoadjuvant immunotherapy, suggesting that VATS is associated with low surgical-related mortality and morbidity and an acceptable conversion rate (17–20). In 2022, Zhang et al. compared VATS and thoracotomy, indicating that VATS achieved comparable surgical-related outcomes, postoperative recovery, and comorbidities to thoracotomy, with the benefit of fewer postoperative intensive care unit (ICU) stays (21). However, VATS exhibited inferiority in lymph node (LN) assessment than thoracotomy. Therefore, debate persists on the optional surgical modality for patients following ICIs treatment.

Robot-assisted thoracoscopic surgery (RATS), an innovative minimally invasive surgical (MIS) technic, was introduced into the thoracic surgery field in 2002 and performed firstly by our team in mainland China in 2009 (22). Nowadays, RATS is gaining increasing interest among thoracic surgeons and has emerged as a viable option for treating NSCLC, serving as a potential alternative to both thoracotomy and VATS (23). RATS offers a high-definitional, magnified, 3-dimensional (3D) visualization, allowing operators to perform complicated surgery precisely, and has a highly flexible mechanical wrist that can maneuver even more efficiently than human hands, providing great convenience in radical lymphadenectomy (24). Compared with VATS, RATS has shown the advantages of increased LN assessment, shorter surgical durations, faster postoperative recoveries, and higher cost-effectiveness for NSCLC patients (24–26). Additionally, RATS may even provide benefits over VATS with reduced conversion risk and blood loss for patients following neoadjuvant therapy (27, 28). However, the research on the safety and feasibility of RATS versus VATS in treating NSCLC patients with neoadjuvant immunochemotherapy is scarce, and the comparison of oncological efficacy has never been reported.

Herein, we compared the perioperative and survival outcomes of NSCLC patients receiving RATS or VATS following neoadjuvant immunochemotherapy, aiming to assess the advantage of RATS over VATS for these patients.

## 2 Materials and methods

### 2.1 Patients

In the present study, we retrospectively reviewed NSCLC patients receiving MIS lobectomy following immunochemotherapy induction from June 2019 to December 2022 at Shanghai Chest Hospital, Shanghai Jiao Tong University School of Medicine. Echocardiography, pulmonary function testing, and electrocardiogram were performed to assess the surgical tolerance of patients. Brain-enhanced magnetic resonance imaging (MRI), positron emission tomography/CT (PET/CT), bone scintigraphy, and abdominal ultrasound were utilized to evaluate distant metastasis. To determine mediastinal LN status, PET/CT was conducted for all patients, and invasive mediastinal assessment, including endobronchial ultrasound (EBUS)-guided transbronchial needle aspiration (TBNA) and mediastinoscopy, were further performed if necessary. All patients were staged by the 8^th^ edition of the American Joint Committee on Cancer (AJCC) Cancer Staging Manual. Before immunochemotherapy induction, the pathological biopsy was performed for all patients, and EGFR (epidermal growth factor receptor) mutation and ALK (anaplastic lymphoma kinase) translocation status were determined. Other oncogene events were tested, if applicable, by adopting next-generation sequencing, fluorescence *in situ* hybridization, or polymerase chain reaction. The preoperative assessment of patients between the two groups was identical.

Eligible patients had a stage IIB to IIIB NSCLC and underwent the simple single lobectomy in a curative intent following neoadjuvant ICIs immunotherapy plus platinum-based doublet chemotherapy. All cases were associated with radiographically measurable lesions following the Response Evaluation Criteria In Solid Tumors (RECIST) version 1.1 and an Eastern Cooperative Oncology Group (ECOG) performance status of 0 or 1. The following were the exclusion criteria (1): cases with missing information (2); clinical N3 stage of the disease (3); intrapulmonary or distant metastasis assessed by preoperative tests.

### 2.2 Therapeutic regimens and treatment

The neoadjuvant regimens and indications for surgery were discussed and determined by a multidisciplinary team (MDT). All patients received PD-1 monoclonal antibodies (Nivolumab, Pembrolizumab, Sintilimab, Tislelizumab, Toripalimab, or Camrelizumab) combined with guideline-recommended platinum-based doublets (pemetrexed, docetaxel, or gemcitabine plus cisplatin or carboplatin), and routinely underwent surgery four to six weeks after the last cycle of therapy. For the selected stage IIIB (T3N2M0) NSCLC patients with single-level N2 involvement, the operation was also performed if an MDT assessment considered they could benefit from surgical resection (29, 30). Nevertheless, cases with initial stage-IV NSCLC who downgraded to the operative clinical stage were excluded due to the controversial oncological efficacy of neoadjuvant immunochemotherapy and various therapeutic approaches to metastatic lesions preoperatively.

### 2.3 Surgical technics

RATS and VATS were performed according to the procedure reported by our surgical team previously (23–25). All patients received general intravenous (*i.v.*) anesthesia with double-lumen intubation and single-lung ventilation managed by dedicated thoracic anesthesiologists. RATS was carried out by adopting the da Vinci Surgical System (Intuitive Surgical, CA, USA) via four minimal incisions of the non-rib spreading technic. The camera port was located on the 7^th^ or 8^th^ intercostal space along the posterior axillary line. Then, two incisions were symmetrically made at the 7^th^ and 9^th^ intercostal spaces along the mid-axillary and infrascapular lines, respectively. A utility port was created at the 3^rd^ or 4^th^ intercostal space on the anterior axillary line for the bedside assistant to expose the operating field, tract lung, and retrieve specimens. Conventionally, VATS was performed via three or four minimal incisions without spreading the ribs. The camera port was created at the 7^th^ intercostal space along the anterior axillary line. Then, two incisions were made at the 3^rd^ or 4^th^, and 8^th^ intercostal spaces on the anterior and posterior axillary lines, respectively. If deemed necessary, a fourth port was created at the 9^th^ intercostal space along the posterior axillary line for assistance. A radical lobectomy with systematic mediastinal LN dissection was carried out for all patients, with the resection margin being evaluated by the intraoperative frozen section. After confirming no air leak and active bleeding, the chest wall was closed with one or two 24F chest tubes placed in the pleural cavity. The conversion was defined as the operation starting with RATS or VATS dissection and finishing as the rib spreading thoracotomy.

### 2.4 Postoperative management and follow up

After operations, patients in the two groups were managed following identical protocols, which included early postoperative activities, breathing training, and specific postoperative analgesia. Dedicated rehabilitation therapists participated throughout the postoperative recovery process for every patient. The decision of ICU administration was made by the surgical team. Generally, the following patients would be treated by ICU after surgery: 1) with life-threaten surgical-related complications; 2) experiencing severe events during operation, such as conversion and blood transfusion; and 3) with the preoperative-assessed baseline potential for serious postoperative comorbidities, for example, elderly, with impaired cardiac or pulmonary function, and with cardiocerebrovascular diseases. The chest tube was removed when the absence of apparent air leak and subcutaneous emphysema was confirmed, the drainage volume of <200 mL/day, no densely bloody, cloudy, or purulent pleural effusion, and the chest X-ray images indicated excellent resorption of the lung. After surgery, patients were routinely evaluated by an MTD and received adjuvant therapy.

The lifelong follow-up assessment was planned one month after the operation, followed by every three months for the first two years, every half year from years three to five, and annually afterward. Thoracic CT scans, abdominal ultrasounds, routine blood tests, and serum tumor marker tests were routinely performed. PET-CT, brain MRI, bone scintigraphy, or TBNA were further applied if deemed necessary. Telephone follow-up was performed every six months until death or May 2023 for patients who did not regularly visit the outpatient clinic. The latest electronic medical profiles were recorded if patients lost to follow-up.

### 2.5 Clinical assessment and outcome measurement

Induction-related adverse events (IRAEs) were assessed in all patients using the National Cancer Institute Common Terminology Criteria for Adverse Events version 4.0 (https://ctep.cancer.gov/), a widely applied grade system for induction-related morbidities (31–33). The thoracic CT scan or PET-CT radiographic assessment was conducted after induction therapy. The radiographic response was determined using RECIST version 1.1 by at least one dedicated thoracic radiologist. Interval to surgery was measured from the end of neoadjuvant treatment to the surgery date, and operation time was measured from incision to wound closure. R0 resection was defined as no microscopic residual tumor confirmed by paraffin pathologic reports. The 30-day postoperative complications were classified per the Clavien-Dindo classification system as follows: grade I, any deviation from the ordinary postoperative course without the need for pharmacological or operational intervention, or merely needing drugs such as analgesics, antipyretics, antiemetics, diuretics, or electrolytes; grade II, complication requiring pharmacological treatment, including blood transfusion and total parenteral nutrition; grade III, comorbidities requiring surgical or endoscopic intervention; grade IV, severe complication requiring ICU treatment; and grade V, death of the patient (34). Specifically, pulmonary comorbidities included pneumonia, acute respiratory distress syndrome (ARDS), respiratory failure requiring reintubation, empyema, and pulmonary embolism. Cardiac comorbidities included arrhythmia and myocardial ischemia or infarction. Anastomotic complications included prolonged air leaks and bronchopleural fistula. Other comorbidities included chylothorax, recurrent laryngeal nerve injury, and wound infection. Pathological response to therapy was evaluated regarding the volume of residual viable tumor cells in relation to the tumor bed following the principle described previously (35). Major pathological response (MPR) was defined as the presence of 10% viable residual tumor cells in the resected specimen, among which pathological complete response (pCR) was indicated when no viable residual tumor cell was found. The PD-L1 status of the tumor cells was assessed, and the positivity was indicated by a tumor proportion score of 1% or more. Recurrence-free survival (RFS) was calculated from the surgy to the date of any local or distant tumor recurrence, while central nervous system (CNS)-free survival was calculated from the surgery to the date of CNS tumor recurrence.

### 2.6 Statistical analysis

Mean ± standard deviation (SD) or median and interquartile range (IQR) were used to express the continuous variables, and frequencies and percentages were applied to define the categorical variables. If the Kolmogorov-Smirnov test indicated a normal distribution and homogeneous variance of the variable, the Student’s t-test was conducted to compare continuous variables. Otherwise, the Mann-Whitney U test was carried out. Pearson’s χ^2^ or Fisher’s exact test was applied to compare categorical variables. Kaplan-Meier curves log-rank (Mantel-Cox) test was adopted to analyze survival profiles. A prespecified two-sided p-value <0.05 was considered statistically significant. IBM SPSS Statistics v.26.0 (IBM Corporation, Armonk, NY, USA) was applied to perform the statistical analysis, while GraphPad Prism 9 (GraphPad Software Inc., San Diego, CA, USA) was adopted to analyze survival profiles.

## 3 Results

### 3.1 Baseline clinical characteristics of patients

**Figure 1:**
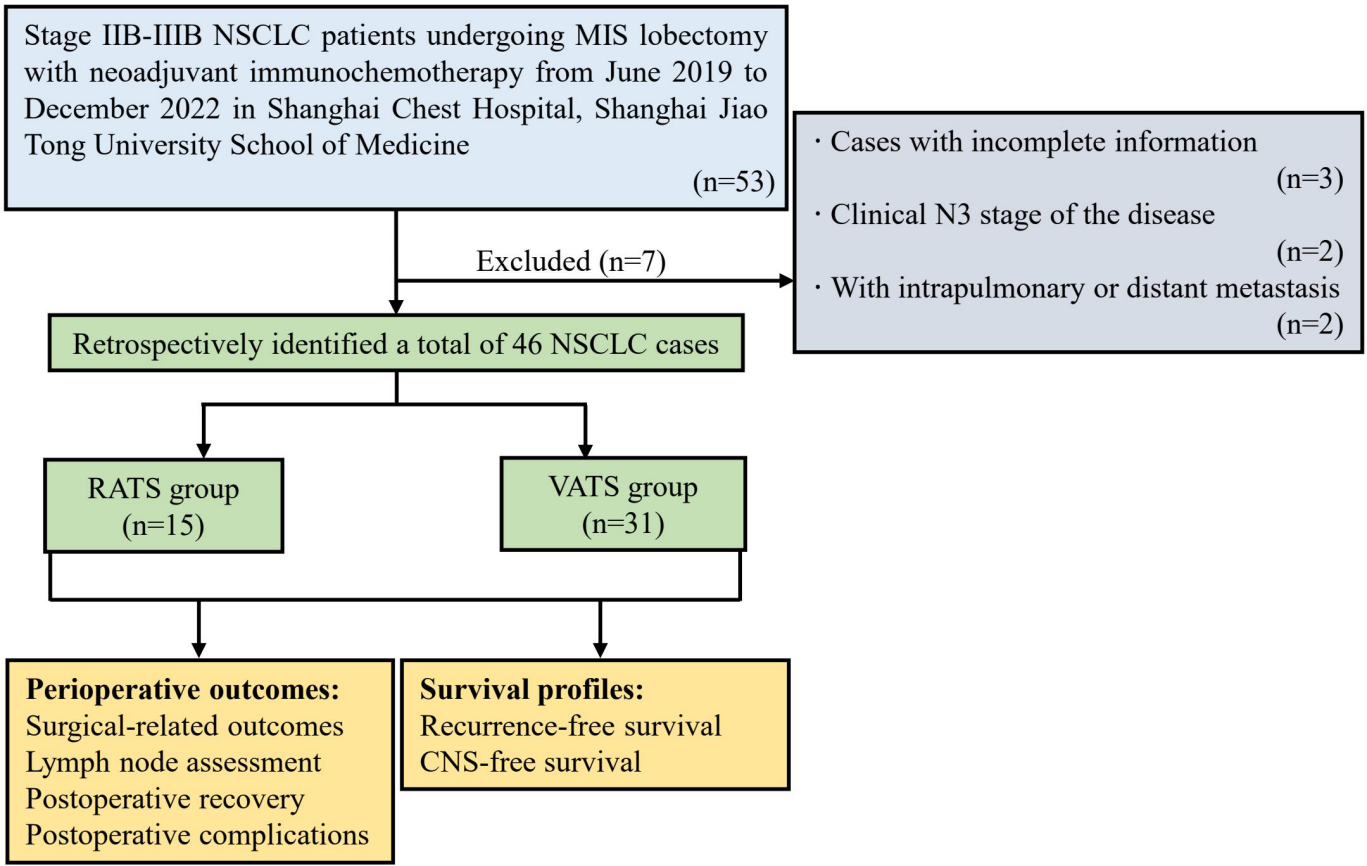
Flow chart of the study population. NSCLC, non-small cell lung cancer; MIS, minimally invasive surgery; RATS, robot-assisted thoracoscopic surgery; VATS, video-assisted thoracoscopic surgery; CNS, central nervous system.

A total of 46 NSCLC patients receiving MISs with neoadjuvant immunochemotherapy were retrospectively identified according to the inclusion and exclusion criteria and then split into RATS (n=15) and VATS (n=31) groups (**Figure 1**). The baseline clinical characteristics of the identified patients are summarized in **Table 1**. The male patients occupied the most dominant in the RATS (93.33%) and VATS (83.37%) groups. The average age of patients receiving RATS and VATS was 61.27 and 61.42 years, respectively. Moreover, 80.00% and 83.87% of cases underwent invasive mediastinal staging in the RATS and VATS groups, respectively. Most participants received three or more neoadjuvant cycles before undergoing RATS (80.00%) or VATS (74.19%). Additionally, patients in the RATS and VATS groups were associated with 53.33% and 54.84% objective radiographic response before the surgery. Finally, the median interval to operation in the RATS and VATS groups were 35.0 and 34.0 days, respectively. By comparison, no significant difference was found between the two groups concerning gender (p=0.647), age (p=0.995), smoking history (p=0.901), presence of comorbidities (p=0.933), BMI (p=0.513), FEV1% (p=0.830), DLCO% (p=0.794), serum albumin level (p=0.644), peripheral white blood cell (WBC) count (p=0.715), tumor anatomic site (p=0.691), tumor location (p=0.603), invasive mediastinal staging rate (p=1.000), tumor histology type (p=0.913), pre-induction TNM stage (p=1.000), neoadjuvant cycle (p=1.000), objective radiographic response rate (p=0.923), and pre-operative TNM stage (p=0.616), and interval to surgery (p=0.723). Given the small sample size and balanced baseline features of included cases, propensity score matching was not further applied.

**Table 1:** Baseline clinical characteristics of patients.

Variables	RATS (n=15)	VATS (n=31)	p*-*value
Gender, n (%)			0.647
Male	14 (93.33)	26 (83.87)	
Female	1 (6.67)	5 (16.13)	
Age, years, mean ± SD	61.27 ± 7.85	61.42 ± 8.14	0.995
Smoking history, n (%)			0.901
Ever	9 (60.00)	18 (58.06)	
Never	6 (40.00)	13 (41.94)	
Comorbidity, n (%)			0.933
Yes	6 (40.00)	12 (38.71)	
No	9 (60.00)	19 (61.29)	
BMI, kg/m^2^, mean ± SD	24.02 ± 2.41	24.40 ± 2.91	0.513
Pulmonary function, mean ± SD			
FEV1 (% of predicted)	94.74 ± 12.61	92.36 ± 11.34	0.83
DLCO (% of predicted)	88.59 ± 26.46	87.49 ± 17.94	0.794
Serum albumin level, g/L, mean ± SD	41.47 ± 3.50	41.42 ± 2.57	0.644
Peripheral WBC count, (×10^9^/L), mean ± SD	6.22 ± 2.03	6.44 ± 1.40	0.715
Invasive mediastinal staging, n (%)	12 (80.00)	26 (83.87)	1
Tumor anatomical site, n (%)			0.691
Right/Left upper lobe	9 (60.00)	22 (70.97)	
Middle lobe	2 (13.33)	2 (6.45)	
Right/Left lower lobe	4 (26.67)	7 (22.58)	
Tumor location, n (%)			0.603
Central	7 (46.67)	17 (54.84)	
Peripheral	8 (53.33)	14 (45.16)	
Tumor histology, n (%)			0.913
Adenocarcinoma	7 (46.67)	15 (48.39)	
Squamous cell carcinoma	8 (53.33)	16 (51.61)	
Pre-induction clinical stage, n (%)			1
IIB	4 (26.67)	8 (25.81)	
IIIA	5 (33.33)	11 (35.48)	
IIIB	6 (40.00)	12 (38.71)	
Neoadjuvant cycles, n (%)			1
On or two	3 (20.00)	8 (25.81)	
Three or more	12 (80.00)	23 (74.19)	
Objective radiographic response, n (%)	8 (53.33)	17 (54.84)	0.923
Pre-operative clinical stage, n (%)			0.616
IA	3 (20.00)	5 (16.13)	
IB	3 (20.00)	3 (9.68)	
IIA	1 (6.67)	1 (3.23)	
IIB	1 (6.67)	7 (22.58)	
IIIA	6 (40.00)	14 (45.16)	
IIIB	1 (6.67)	1 (3.23)	
Interval to surgery, day, median [IQR]	35.0 [27.0-40.5]	34.0 [29.5-39.5]	0.723

Continuous data are expressed as mean ± SD or median [IQR], and categorical data are shown as number (percentage). RATS, robot-assisted thoracoscopic surgery; VATS, video-assisted thoracoscopic surgery; SD, standard deviation; BMI, body mass index; FEV1, forced expiratory volume in 1 s; DLCO, diffusing capacity for carbon monoxide; IQR, interquartile range.

### 3.2 Neoadjuvant treatment-related adverse events

The induction toxicity in the preoperative setting is expressed in **Table 2**. Overall, the proportion of patients with IRAEs in the RATS and VATS groups was 53.33% (8 of 15 patients) and 54.84% (17 of 31 patients), respectively. Altogether, the most frequent IRAEs in the two groups were neutropenia (30.43%), anemia (28.26%), increased aminotransferases (15.22%), rash (15.22%), and peripheral sensory neuropathy (13.04%), with all the grade-4 IRAEs being myelosuppression (6.52%). By comparison, the induction-related AEs were similar between the RATS and VATS groups (all p>0.050). All IRAEs were manageable with symptomatic treatment or observation only.

**Table 2:** Neoadjuvant treatment-related adverse events of patients.

Variables	RATS (n=15)	VATS (n=31)	p*-*value
Any IRAEs, n (%)	8 (53.33)	17 (54.84)	0.923
Neutropenia, n (%)			1
Grade 1-2	3 (20.00)	7 (22.58)	
Grade 3-4	1 (6.67)	3 (9.68)	
Anemia, n (%)			1
Grade 1-2	3 (20.00)	7 (22.58)	
Grade 3	1 (6.67)	2 (6.45)	
Increased aminotransferases, n (%)			1
Grade 1-2	2 (13.33)	4 (12.90)	
Grade 3	0 (0.00)	1 (3.23)	
Rash, n (%)			1
Grade 1-2	2 (13.33)	4 (12.90)	
Grade 3	0 (0.00)	1 (3.23)	
Peripheral sensory neuropathy, n (%)			1
Grade 1-2	2 (13.33)	4 (12.90)	
Grade 3	0 (0.00)	0 (0.00)	
Fatigue, n (%)			1
Grade 1-2	2 (13.33)	3 (9.68)	
Grade 3	0 (0.00)	0 (0.00)	
Nausea, n (%)			1
Grade 1-2	2 (13.33)	3 (9.68)	
Grade 3	0 (0.00)	0 (0.00)	
Anorexia, n (%)			0.587
Grade 1-2	2 (13.33)	2 (6.45)	
Grade 3	0 (0.00)	0 (0.00)	
Arthralgia or myalgia, n (%)			1
Grade 1-2	1 (6.67)	3 (9.68)	
Grade 3	0 (0.00)	0 (0.00)	
Alopecia, n (%)			1
Grade 1-2	1 (6.67)	2 (6.45)	
Grade 3	0 (0.00)	0 (0.00)	
Pruritus, n (%)			1
Grade 1-2	1 (6.67)	1 (3.23)	
Grade 3	0 (0.00)	0 (0.00)	
Thrombocytopenia, n (%)			0.541
Grade 1-2	0 (0.00)	3 (9.68)	
Grade 3	0 (0.00)	0 (0.00)	
Hypothyroidism, n (%)			0.326
Grade 1-2	1 (6.67)	0 (0.00)	
Grade 3	0 (0.00)	0 (0.00)	

Data are shown as number (percentage). RATS, robot-assisted thoracoscopic surgery; VATS, video-assisted thoracoscopic surgery; IRAEs, induction-related adverse events.

### 3.3 Surgical outcomes, pathological results, and LN assessment

As expressed in **Table 3**, RATS and VATS led to comparable operative time (159.27 ± 26.77 vs. 171.65 ± 42.05 min, p=0.483), R0 resection rate (93.33% vs. 93.55%, p=1.000), and incidence of conversion to thoracotomy (6.67% vs. 9.68%, p=1.000). Additionally, blood transfusion was not required in patients undergoing RATS, while was performed for one and two patients receiving VATS intraoperatively and postoperatively, respectively. By comparison, patients in the RATS and VATS groups were associated with similar intraoperative bleeding (p=0.421) and incidence of intraoperative (p=1.000) and postoperative blood transfusion (p=1.000).

**Table 3:** Surgical outcomes and postoperative recoveries of patients.

Variables	RATS (n=15)	VATS (n=31)	p*-*value
Operative time, min, mean ± SD	159.27 ± 26.77	171.65 ± 42.05	0.483
R0 resection, n (%)	14 (93.33)	29 (93.55)	1
Conversion to thoracotomy, n (%)	1 (6.67)	3 (9.68)	1
Intraoperative bleeding, mL, n (%)			
≤100	11 (73.33)	19 (61.29)	0.421
>100	4 (26.67)	12 (38.71)	
Blood transfusion, n (%)			
Intraoperative	0 (0.00)	1 (3.23)	1
Postoperative	0 (0.00)	2 (6.45)	1
ICU			
Non-surgical-related admission, n (%)	3 (20.00)	9 (29.03)	0.723
Readmission, n (%)	0 (0.00)	3 (9.68)	0.541
Length of surgical-related stay, day, median [IQR]	0.0 [0.0-0.0]	0.0 [0.0-1.0]	0.026
Postoperative chest tube drainage, median [IQR]			
Volume, mL	1270.0 [785.0-1755.0]	1440.0 [860.0-1885.0]	0.578
Duration, day	5.0 [3.5-7.0]	6.0 [5.0-7.0]	0.507
Postoperative hospital stay, day, median [IQR]	6.0 [4.5-8.0]	7.0 [6.0-8.0]	0.802

Continuous data are expressed as mean ± SD or median [IQR], and categorical data are shown as number (percentage). RATS, robot-assisted thoracoscopic surgery; VATS, video-assisted thoracoscopic surgery; SD, standard deviation; IQR, interquartile range; ICU, intensive care unit; LN, lymph node; IPR, incomplete pathological response; MPR, major pathological response; pCR, pathological complete response; yP, yield pathological.

**Figure 2:**
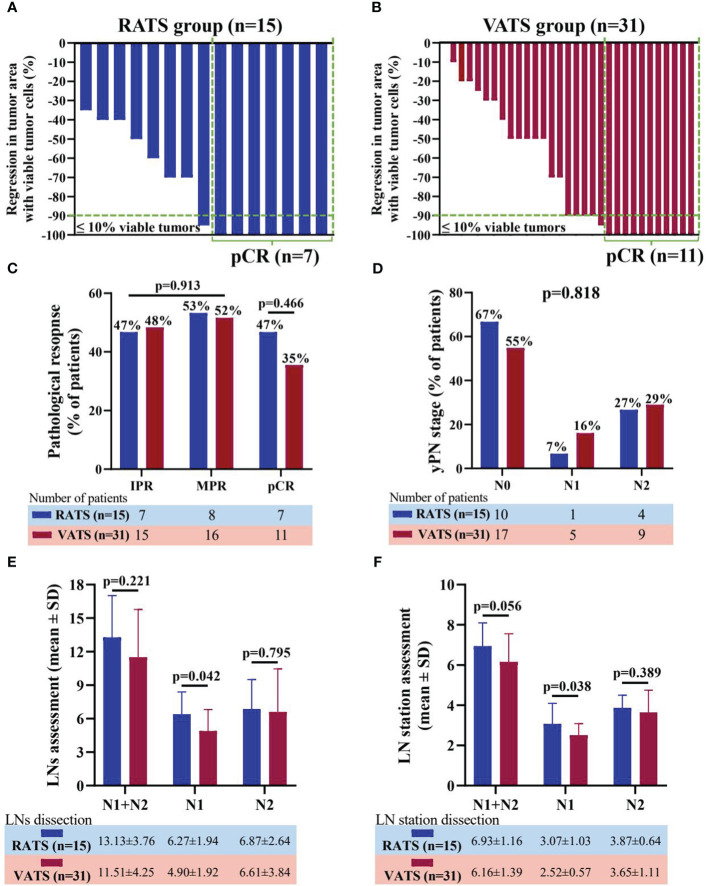
Waterfall plots of regression in tumor area with viable tumor cells in resection specimen of patients receiving RATS **(A)** or VATS **(B)** following neoadjuvant immunochemotherapy. Each bar represents one patient. Comparison of pathological response and pCR rate **(C)** and yPN stage **(D)** between the RATS and VATS groups. Comparison of dissected LNs **(E)** and LN stations **(F)** between the RATS and VATS groups. NSCLC, non-small cell lung cancer; RATS, robot-assisted thoracoscopic surgery; VATS, video-assisted thoracoscopic surgery; IPR, incomplete pathological response; MPR, major pathological response; pCR, pathological complete response; yP, yield pathological; LNs, lymph nodes.

Additionally, the regression in tumor area with viable tumor cells in resection specimens of the individual patient undergoing RATS or VATS was shown in **Figures 2A, B**. Overall, in the RATS and VATS groups, the MPR rates of patients were 53.33% (8 of 15 patients) and 51.61% (16 of 31 patients), respectively, and the pCR rates were 46.67% (7 of 15 cases) and 35.48% (11 of 31 cases), respectively (**Figure 2C**). By comparison, there was no difference in the two groups concerning the pathological response (p=0.913) and pCR rate (p=0.466). Finally, RATS and VATS achieved the comparable ypN stage (p=0.818, **Figure 2D**).

In terms of LN dissection, RATS assessed significantly increased N1 LNs (6.27 ± 1.94 vs. 4.90 ± 1.92, p=0.042) and stations (3.07 ± 1.03 vs. 2.52 ± 0.57, p=0.038) than VATS (**Figure 2E, F**). Nevertheless, RATS and VATS were comparable in harvesting N2 (6.87 ± 2.64 vs 6.61 ± 3.84, p=0.795) and total LNs (13.13 ± 3.76 vs 11.51 ± 4.25, p=0.221), and N2 (3.87 ± 0.64 vs 3.65 ± 1.11, p=0.389) and total LN stations (6.93 ± 1.16 vs 6.16 ± 1.39, p=0.056).

### 3.4 Postoperative recovery and surgical-related complications

As expressed in **Table 3**, RATS reduced the duration of surgical-related ICU stay compared with VATS (0.0 [0.0-0.0] vs. 0.0 [0.0-1.0] days, p=0.026). Meanwhile, the two groups had a comparable incidence of ICU readmission (0.00% vs. 9.68%, p=0.541) and non-surgical-related admission (20.00% vs. 29.03%, p=0.723). Additionally, patients in the RATS and VATS groups were associated with similar postoperative chest tube drainage volume (1270.0 [785.0-1755.0] vs. 1440.0 [860.0-1885.0] mL, p=0.578) and duration (5.0 [3.5-7.0] vs. 6.0 [5.0-7.0] days, p=0.507), and postoperative hospital stay (6.0 [4.5-8.0] vs. 7.0 [6.0-8.0] days, p=0.802).

**Figure 3:**
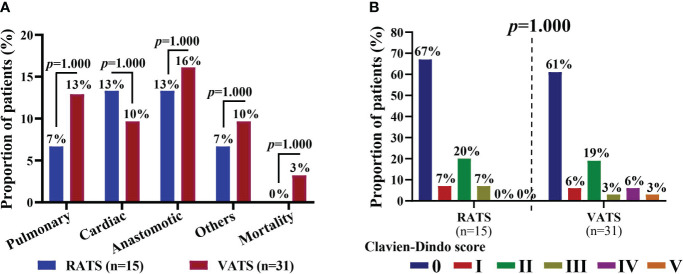
**(A)** Distribution of 30-day postoperative complications of NSCLC patients in the RATS and VATS groups. Patients may be associate with multiple (≥2) complications. **(B)** Comparison of Clavien-Dindo scores of NSCLC patients in the RATS and VATS groups. The highest grade was indicated if a patient was associated multiple comorbidities. NSCLC, non-small cell lung cancer; RATS, robot-assisted thoracoscopic surgery; VATS, video-assisted thoracoscopic surgery.

The postoperative complications of patients are shown in **Table 4**. Five and twelve patients had postoperative complications in the RATS and VATS groups, respectively, and one and three had severe comorbidities (Clavien-Dindo score ≥3). Surgical-related mortality was not occurred in the RATS groups, while one patient died within 30 days after VATS. Overall, prolonged air leak, arrhythmia, and pneumonia were the most common complications in patients receiving MISs following neoadjuvant immunochemotherapy. By comparison, RATS and VATS led to comparable incidences of overall (33.33% vs. 38.71%, p=0.723) and severe complications (6.47% vs. 12.90%, p=1.000) and any individual comorbidities (all p>0.050). Additionally, the distribution of complications was similar in the two groups (all p>0.050, **Figure 3A**). Finally, patients in the RATS and VATS groups were associated with an equal distribution of 30-day Clavien-Dindo postoperative complication scores (p=1.000, **Figure 3B**).

**Table 4:** Postoperative complications of patients.

Variables	RATS (n=15)	VATS (n=31)	p*-*value
Any complications, n (%)	5 (33.33)	12 (38.71)	0.723
Severe complications^a^	1 (6.67)	4 (12.90)	1
Pulmonary complications, n (%)			
Pneumonia	1 (6.67)	3 (9.68)	1
Respiratory failure requiring reintubation	0 (0.00)	2 (6.45)	1
Empyema	0 (0.00)	1 (3.23)	1
Cardiac complications, n (%)			
Arrhythmia	2 (13.33)	3 (9.68)	1
Anastomotic complications, n (%)			
Prolonged air leak >5 days	2 (13.33)	5 (16.13)	1
Bronchopleural fistula	0 (0.00)	2 (6.45)	1
Other complications, n (%)			
Hemorrhage requiring intervention	0 (0.00)	2 (6.45)	1
Chylothorax	1 (6.67)	1 (3.23)	1
Readmission within 30 days, n (%)	0 (0.00)	2 (6.45)	1
30-Day mortality, n (%)	0 (0.00)	1 (3.23)	1

Categorical data are shown as number (percentage). ^a^Comorbidities with Clavien-Dindo score ≥3. RATS, robot-assisted thoracoscopic surgery; VATS, video-assisted thoracoscopic surgery.

### 3.5 Survival outcomes

**Figure 4:**
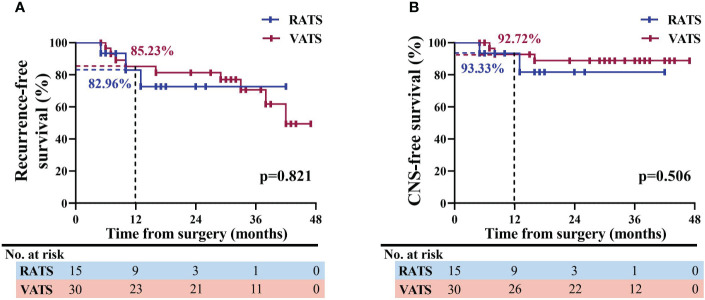
Analysis of survival profiles of NSCLC patients receiving RATS or VATS following neoadjuvant immunochemotherapy. Kaplan-Meier comparison of RFS **(A)** and CNS-free survival **(B)** profiles between the RATS and VATS groups. NSCLC, non-small cell lung cancer; RATS, robotic-assisted thoracoscopic surgery; VATS, video-assisted thoracoscopic surgery; RFS, recurrence-free survival; CNS, central nervous system.

RFS is deemed an indicator of oncological efficacy, and therefore one patient who experienced surgical-related mortality in the VATS group was excluded when evaluating RFS. As shown in **Figure 4**, in a median follow-up time of 26.5 months [IQR, 16.0-37.0 months], patients in the RATS and VATS groups were associated with comparable one-year RFS profiles (82.96% vs. 85.23% months, p=0.821, **Figure 4A**). The swimming plot of survival profiles is presented in **Figure S1**. By the additional comparison, no significant difference was found between RATS and VATS considering the one-year CNS-free (93.33% vs. 92.72%, p=0.506, **Figure 4B**), LN-free (88.89% vs. 89.01%, p=0.661, **Figure S2A**), and bone-free (87.50% vs. 92.98%, p=0.937, **Figure S2B**) survivals.

## 4 Discussion

Nowadays, neoadjuvant immunochemotherapy has revolutionized the treatment of NSCLC, providing dramatic survival benefits compared with traditional chemotherapy. Although induction therapy usually adds surgical difficulty and perioperative challenges, VATS is becoming increasingly prevalent and has been considered safe and feasible in treating NSCLC patients following neoadjuvant immunochemotherapy (18, 21). Nevertheless, the application of RATS, an innovative MIS technic offering perioperative and even survival improvement than VATS for early-stage NSCLC, in treating patients with neoadjuvant immunochemotherapy has been rarely reported (25, 36–38). Consequently, research on the safety, feasibility, and oncological efficacy of RATS versus VATS for these patients is scarce. In the present study, we compared short-term outcomes of RATS versus VATS for NSCLC patients following immunochemotherapy, indicating that RATS exhibited superiorities in reducing postoperative ICU stay and dissecting more N1 LNs and stations, though achieving similar survival profiles to VATS.

With regard to surgical-related outcomes, our results showed that RATS led to a 6.67% of conversion rate, which was comparable to VATS (9.68%), and thus both surgical methods appear to be feasible with acceptable conversion rates. This conclusion is aligned with many previous publications enrolling early-stage or locally advanced NSCLC cases (23, 24, 39). However, at least four research revealed that fewer patients who received RATS converted to thoracotomy when compared with those undergoing VATS (27, 40–42). Importantly, RATS was also found to reduce conversion incidence for NSCLC patients with neoadjuvant immunochemotherapy than VATS (43). We notice that in our study, the conversion rate in NSCLC patients undergoing RATS after neoadjuvant immunochemotherapy was consistent with the previous ones, which ranged from 4.5% to 7% (43, 44). Nevertheless, it is much lower in patients receiving VATS in our study (9.68%) than that reported by previous publications ranging from 19% to over 50% (16, 20, 21, 45). Therefore, the controversial conclusion comparing the conversion incidence of RATS and VATS between our study and previous ones may largely be attributed to the decreased conversion rate of VATS. Given this, a high-volume medical center may reduce the risk of conversion of VATS for NSCLC patients following neoadjuvant immunochemotherapy. Finally, RATS and VATS achieved excellent bleeding management, with most of the patients having blood loss of less than 100 mL, and only one patient in the VATS group required intraoperative blood transfusion. For these reasons, both approaches seem to be safe concerning bleeding control.

In our study, RATS significantly reduced the length of surgical-related ICU stay than VATS. This superiority of RATS is potentially due to the great flexibility of the robot arms and high-quality surgical view that enable thoracic surgeons to perform resection and LN retrieval more precisely and thus minimize unnecessary damage to normal tissues, especially for patients with adhesive, fibrotic, and brittle tissues and fibro-calcified LNs caused by induction therapy, which accelerated the patient recovery (39, 43). Moreover, the fewer surgical-related complications requiring ICU administration in the RATS group than VATS (0.00% vs 9.68%), despite no statistical significance being found, could also contribute to the reduction in ICU stay. Nevertheless, the faster recovery from ICU in the RATS group did not dramatically lead to a more immediate discharge, although RATS appeared to be associated with a shorter chest tube duration and postoperative hospitalization to VATS. This might be attributed to the small sample size of cases in the present study. Given this, further study based on a larger cohort of patients is necessary further to compare the postoperative recoveries between the two approaches.

LN assessment is pivotal for the surgical treatment of NSCLC and a critical standard measuring the operative quality, and concerns about LN dissection have commonly been a drawback for VATS in treating NSCLC patients, especially those with the involved LNs or neoadjuvant therapy (21, 24, 46). Many previous publications have reported the comparison of LNs and LN station assessment between RATS and VAST but have drawn conflicting conclusions. At least two large-scale retrospective studies have revealed that RATS could dissect more LNs than VATS, which was further verified by two clinical trials reported recently (39, 47–49). However, several other research did not observe this superiority of RATS over VATS (50–52). In the present study, RATS dissected more N1 LNs and stations than VATS, which was in line with a previous publication, suggesting that RATS may possess the superiority over VATS in LN assessment for NSCLC patients with neoadjuvant immunochemotherapy (43). This is primarily attributed to the surgical view with 3D, high-definition, and ten-fold magnification and robotic arms with excellent maneuverability and improved dexterity provided by the robotic-assisted surgical system that offers operators great convenience to harvest LNs around vessels and bronchi (24, 39). Additionally, our results showed that RATS did assess an increased number of N2 and total LNs and LN stations than VATS. However, no statistical difference was found, which might be attributed to the small sample size included, and thus a larger cohort is necessary to validate our conclusion. Moreover, our study indicated that the increased LN assessment by RATS over VATS was not correlated with higher ypN upstaging in our study, which might be attributed to the good LN response to neoadjuvant immunochemotherapy and more than half of patients being associated with the stage ypN0 disease, as well as the comparable N2 LNs and stations assessed by the two approaches.

Although the increased examined LNs might lead to a more thorough elimination of remnants, its correlation to potential survival benefits remains controversial. At least four research found that an increased LN dissection contributed to prolonged survival, and at least 10 LNs should be harvested, while extra assessment of more than 16 LNs did not lead to better oncological outcomes (53–56). However, three others did not find this association (21, 57, 58). Moreover, many studies have found increased capability of RATS in LN assessment, but none of them observed its superiority in survival profiles over VTAS, which is consistent with our results (24, 25, 59, 60). Until now, the correlation between LN dissection and survival for NSCLC patients with neoadjuvant immunochemotherapy remains unrevealed, and further research is needed. Previous studies have found that lymphocytes in tumor-drainage LNs exhibit robust anti-tumor efficacies, and their activation and cytotoxicity effects upon immunotherapy dramatically improve survival in NSCLC patients (61, 62). Given this, the remaining LNs may also enhance the therapeutic efficacy of adjuvant immunotherapy ± chemotherapy to eliminate for patients receiving surgery following neoadjuvant ICI treatment. Paradoxically, the deficient LN dissection could lead to undiscovered metastatic LNs, disrupting the therapy and ultimately resulting in recurrence and distant dissemination (63). Therefore, the adequate LN examination and the preservation of the regional immune microenvironment should be balanced, and the optimal number of examined LNs and LN stations for NSCLC patients with neoadjuvant immunotherapy requires further investigation.

In terms of survival outcomes, our results showed that RATS and VATS achieved comparable 1-year RFS and CNS-free survival, and thus the two approaches appear to have similar oncological efficacies. In previous studies, RATS and VATS usually achieved comparable long-term outcomes in treating NSCLC (24, 41, 64). These conclusions, together with ours, indicated that the approach of MISs may not impact the long-term survival of NSCLC patients. Nevertheless, RATS may be associated with less long-term postoperative pain, improved life quality, and better mental health than VATS for NSCLC patients (64–66). Since neoadjuvant immunochemotherapy could dramatically prolong the survival period of patients with locally advanced NSCLC, evaluating their postoperative life qualities is necessary. We are now performing further follow-ups to compare the oncological and life-quality effectiveness of RATS versus VATS for NSCLC patients with neoadjuvant immunochemotherapy.

Previous studies have revealed that the robot-assisted surgical system could be adapted to perform highly difficult thoracic surgeries, including sleeve or double-sleeve resection for centrally located NSCLC (67–69). Nowadays, sleeve lobectomy has become a preferred surgical approach for centrally located NSCLC due to its reduced postoperative morbidity, lower mortality, better long-term survival and quality of life, and comparable oncological efficacy compared to pneumonectomy (70–72). More importantly, sleeve lobectomy has also proven feasible and oncologically effective for NSCLC patients following chemotherapy or immunochemotherapy induction (32, 73). Therefore, further evaluation of RATS for sleeve resection following neoadjuvant immunochemotherapy is necessary to expand its application for centrally located NSCLC patients.

We have acknowledged some limitations of our research. First, the sample size of our study was small, resulting in many negative results, and PSM was not further applied. These could have influenced the data validity and prejudiced the representative of the results. Secondly, the retrospective nature of the present study may result in undiscovered patient selection bias, despite the comparable baseline clinical features between the RATS and VATS groups. Finally, the long-term outcomes were unavailable due to the relatively short period of MIS’s application for NSCLC patients following neoadjuvant immunochemotherapy. Therefore, the multi-center, prospective study enrolling a more significant number of cases is necessary to confirm the conclusion of our research, and further follow-up is needed to analyze the oncological efficacy of RATS versus VATS. Nevertheless, MIS is likely to be increasingly applied with neoadjuvant immunochemotherapy due to its potential advantages over thoracotomy for NSCLC patients after induction therapy, though it may require a high surgical technic of thoracic surgeons (14, 21, 46, 74, 75). Thus, we believe that it is necessary to compare RATS and VATS, two pivotal MIS technics, for these patients based on the current practice, which might provide a reference for thoracic surgeons in further research and clinical practice.

## 5 Conclusion

In conclusion, RATS is a safe and feasible approach in treating NSCLC patients following neoadjuvant immunochemotherapy, exhibiting superiorities over VATS in shortening postoperative ICU stay and assessing increased N1 LNs and stations, though the two surgical approaches achieved similar survival profiles.

## Data availability statement

The raw data supporting the conclusions of this article will be made available by the authors, without undue reservation.

## Ethics statement

The studies involving human participants were reviewed and approved by The Institutional Review Board of Shanghai Chest Hospital, Shanghai Jiao Tong University School of Medicine (approval number: IS23017). Written informed consent for participation was not required for this study in accordance with the national legislation and the institutional requirements.

## Author contributions

Conception and design: LJ, JH, and QL. Resources: NZ, YT, ZG, HZ, ZL, and LJ. Methodology: HP, NZ, YT, JN, and WJ. Software: YT and WJ. Formal analysis: HP, NZ, JZ. Investigation: HP, NZ, YT, ZG, JZ, WK. Validation: HP, NZ. Visualization: HP, NZ, and YT. Writing-original draft: HP, NZ, and YT. Writing-review & editing: ZL, LJ, JH, and QL. Supervision: JH, and QL. Data curation: JH and QL. Project administration: QL. Funding acquisition: QL. All authors contributed to the article and approved the submitted version.
