# Supplementary material for: Short-term outcomes of robot-assisted versus video-assisted thoracoscopic surgery for non-small cell lung cancer patients with neoadjuvant immunochemotherapy: a single-center retrospective study
Source: Front Immunol. 2023 Jul 11;14:1228451. doi: 10.3389/fimmu.2023.1228451 (PMC10366598; doi:10.3389/fimmu.2023.1228451)
Supplement: Supplementary file 1 [file DataSheet_1.docx]

**Supplementary materials**

**Short-term outcomes of robot-assisted versus video-assisted thoracoscopic surgery** **for non-small cell lung cancer patients with neoadjuvant immunochemotherapy: a single-center retrospective study**

**Hanbo Pan ^1†^, Ningyuan Zou ^1†^, Yu Tian ^1†^, Hongda Zhu ^1^, Jiaqi Zhang ^1^, Weiqiu Jin ^1^, Zenan Gu ^1^, Junwei Ning ^1,2^, Ziming Li ^3^, Weicheng Kong ^1,4^, Long Jiang ^1^, Jia Huang ^1*^, Qingquan Luo ^1*^**

^1^ Department of Thoracic Surgical Oncology, Shanghai Lung Cancer Center, Shanghai Chest Hospital, Shanghai Jiao Tong University School of Medicine, Shanghai, China

^2^ Department of Thoracic Surgery, Shanghai Tongren Hospital, Shanghai Jiao Tong University School of Medicine, Shanghai, China

^3^ Department of Oncology, Shanghai Lung Cancer Center, Shanghai Chest Hospital, Shanghai Jiao Tong University School of Medicine, Shanghai, China

^4^ Department of Thoracic Surgery, Putuo District People’s Hospital, Zhejiang, China

^†^ These authors contributed equally to this work and share the first authorship.

**^*^ Correspondence**

**Qingquan Luo**

E-mail: [luoqingquan@hotmail.com](mailto:luoqingquan@hotmail.com).

**Jia Huang**

E-mail: [huangjiadragon@126.com](mailto:huangjiadragon@126.com).

**
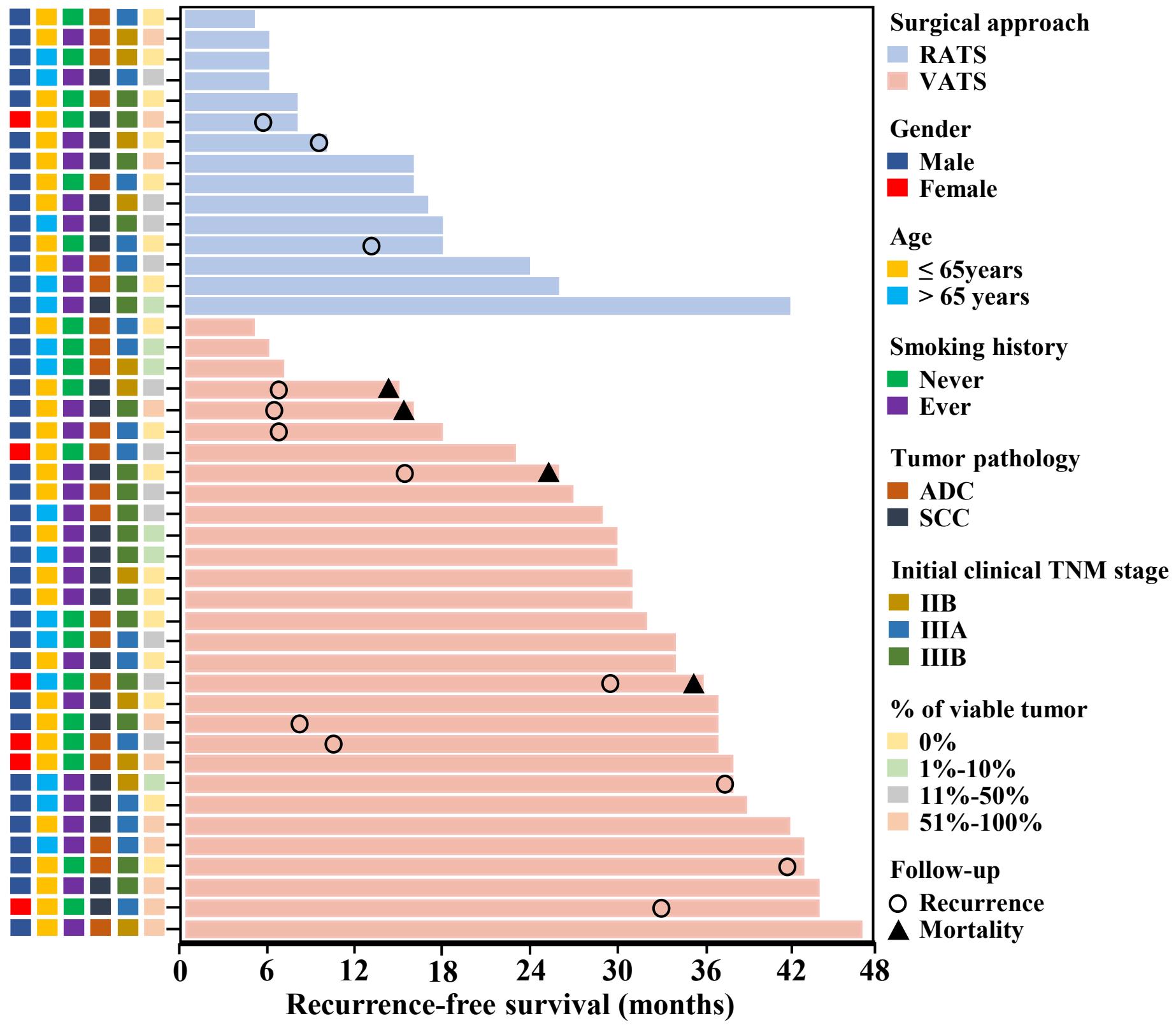
**

**FIGURE S1.** Swimming plot of survival profiles of NSCLC patients who underwent RATS or VATS following neoadjuvant immunochemotherapy. Each bar represents one patient. The left column presents clinical characteristics. At the date cutoff 1^st^ May 2023, 13 of 46 (28.26%) patients were associated with recurrence, of whom 4 (8.70%) died. *NSCLC, non-small cell lung cancer; RATS, robotic-assisted thoracoscopic surgery; VATS, video-assisted thoracoscopic surgery.*

**
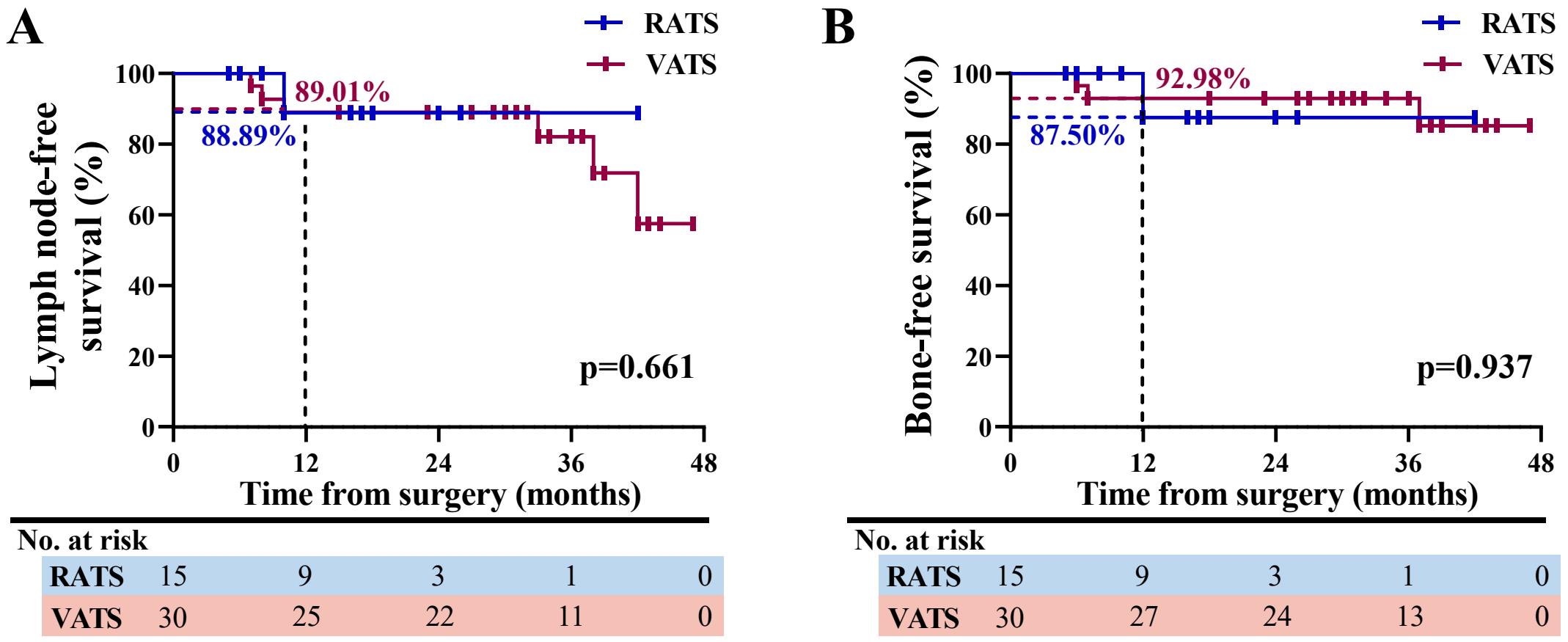
**

**FIGURE S2.** Kaplan-Meier analysis of survival profiles of NSCLC patients receiving RATS or VATS following neoadjuvant immunochemotherapy. Comparison of lymph node-free survival (**A**) and bone-free survival (**B**) profiles between the RATS and VATS groups. *NSCLC, non-small cell lung cancer; RATS, robotic-assisted thoracoscopic surgery; VATS, video-assisted thoracoscopic surgery.*
